# Designing Inhibitors of M2 Proton Channel against H1N1 Swine Influenza Virus

**DOI:** 10.1371/journal.pone.0009388

**Published:** 2010-02-23

**Authors:** Qi-Shi Du, Ri-Bo Huang, Shu-Qing Wang, Kuo-Chen Chou

**Affiliations:** 1 Guangxi Academy of Sciences, Nanning, Guangxi, China; 2 College of Life Science and Technique, Guangxi University, Nanning, Guangxi, China; 3 School of Pharmaceutical Sciences, Tianjin Medical University, Tianjin, China; 4 Gordon Life Science Institute, San Diego, California, United States of America; University of Delhi, India

## Abstract

**Background:**

M2 proton channel of H1N1 influenza A virus is the target protein of anti-flu drugs amantadine and rimantadine. However, the two once powerful adamantane-based drugs lost their 90% bioactivity because of mutations of virus in recent twenty years. The NMR structure of the M2 channel protein determined by Schnell and Chou (Nature, 2008, 451, 591–595) may help people to solve the drug-resistant problem and develop more powerful new drugs against H1N1 influenza virus.

**Methodology:**

Docking calculation is performed to build the complex structure between receptor M2 proton channel and ligands, including existing drugs amantadine and rimantadine, and two newly designed inhibitors. The computer-aided drug design methods are used to calculate the binding free energies, with the computational biology techniques to analyze the interactions between M2 proton channel and adamantine-based inhibitors.

**Conclusions:**

1) The NMR structure of M2 proton channel provides a reliable structural basis for rational drug design against influenza virus. 2) The channel gating mechanism and the inhibiting mechanism of M2 proton channel, revealed by the NMR structure of M2 proton channel, provides the new ideas for channel inhibitor design. 3) The newly designed adamantane-based inhibitors based on the modeled structure of H1N1-M2 proton channel have two pharmacophore groups, which act like a “barrel hoop”, holding two adjacent helices of the H1N1-M2 tetramer through the two pharmacophore groups outside the channel. 4) The inhibitors with such binding mechanism may overcome the drug resistance problem of influenza A virus to the adamantane-based drugs.

## Introduction

Recently, the outbreak of H1N1 influenza A virus is a pandemic of a new strain of influenza virus [1] identified in April 2009, commonly referred to as “swine flu”. Within only four months, the pandemic has caused many deaths from the first detected country Mexico to almost all countries of the world (http://www.who.int/csr/disease/swineflu/). The H1N1 influenza virus is quite familiar to us because it had caused the 1918–1919 Spain pandemic that had infected 5% of the world population and resulted in 20–50 million deaths worldwide [1]. In July 2009 the WHO (World Health Organization) enhanced the warning to phase 6, meaning that the spread of H1N1 influenza virus has become a serious global pandemic. It was anticipated that a stronger outbreak might occur in the coming winter. The even worse news is that cases were reported that several strains of H1N1 influenza A viruses were resistant to oseltamivir (Tamiflu).

Although an influenza virus only possesses eight genes (far less than the estimated 25,000 that a human being has), its simplicity has not stopped it from wreaking havoc on human beings for centuries. “The only thing predictable about influenza is its unpredictability” [2]. Influenza A virus has the ability to undergo changes by the mechanisms of antigenic drift and shift, resulting in new evolving virus strains, which may be extremely toxic and drug-resistant [3]–[5]. Given that influenza shifts may occur every 20–30 years, the danger of future influenza A pandemics highlights the need to develop more effective drugs. The threat of an impending influenza pandemic, possibly through the mutations of the present avian strain H5N1 or swine strain H1N1, has triggered a global effort to develop more effective antivirus drugs. However, during the past several decades many efforts in developing anti influenza drugs have almost been futile due to the rapid mutations of the influenza virus, resulting in the persistent resistance to the existing drugs.

The M2 protein [6]–[9] from influenza A virus is a pH-sensitive proton channel that mediates acidification of the interior of viral particles entrapped and replication in endosomes [10]. Since the M2 protein was found, it has been the main target for finding drugs against influenza A virus. The adamantane-based drugs, amantadine and rimantadine [11]–[13], which target the M2 channel, had been used for many years as the first-choice antiviral drugs against community outbreaks of influenza A viruses. However, the once powerful drugs lost their effectivity quickly due to mutations and evolutions of influenza A viruses. Recent reports show that the resistance of influenza A virus to the adamantane-based drugs in humans, birds and pigs has reached more than 90% [3], [4].

To solve the drug-resistance problem, a reliable molecular structure of M2 proton channel is absolutely necessary [14], [15]. Very recently, using high-resolution nuclear magnetic resonance (NMR) spectroscopy, Schnell and Chou [16] for the first time successfully determined the solution structure of M2 proton channel. They reported an unexpected mechanism of its inhibition by the flu-fighting adamantane drug family. According to the novel mechanism, rimantadine binds at four equivalent sites near the “tryptophan gate” on the lipid-facing side of the channel and stabilizes the closed conformation of the pore. This is completely different from the traditional view but more reasonable in the sense of energetics [17]–[19].

The new discovery of M2 proton channel structure has brought us the light, by which the drug-resistance problem may be solved, and more powerful adamantine-based drugs may be developed. This is because if we can understand how the drug blocks the channel and how mutations evade the effect of the drug, we can come up with better approaches to block it [20].

Based on such a rationale as well as the high-resolution NMR structure of M2 proton channel [16], the present study was initiated in an attempt to solve the drug resistant problem and to design more effective adamantine-based drugs by conducting molecular modeling and docking studies.

## Materials and Methods

Until September 4, 2009, a total of 48 amino acid sequences of H1N1-M2 proton channel proteins are deposited in the website NCBI (www.ncbi.nlm.nih.gov). However, no experimental 3D (three-dimensional) structure of H1N1-M2 protein is reported so far. To develop its 3D structure, the H1N1-M2 protein sequence with the NCBI code of GQ385303 (www.ncbi.nlm.nih.gov) is used in this study. The sequence is isolated in Toronto from a H1N1 virus strain in July 2009. To build the three dimensional structure of GQ385303, the high-resolution NMR structure of M2 proton channel [16] with the PDB code of 2RLF (www.rcsb.org/pdb) was adopted as a template, which was determined for the M2 channel isolated from the Udorn strain of human influenza virus.

**Figure 1:**
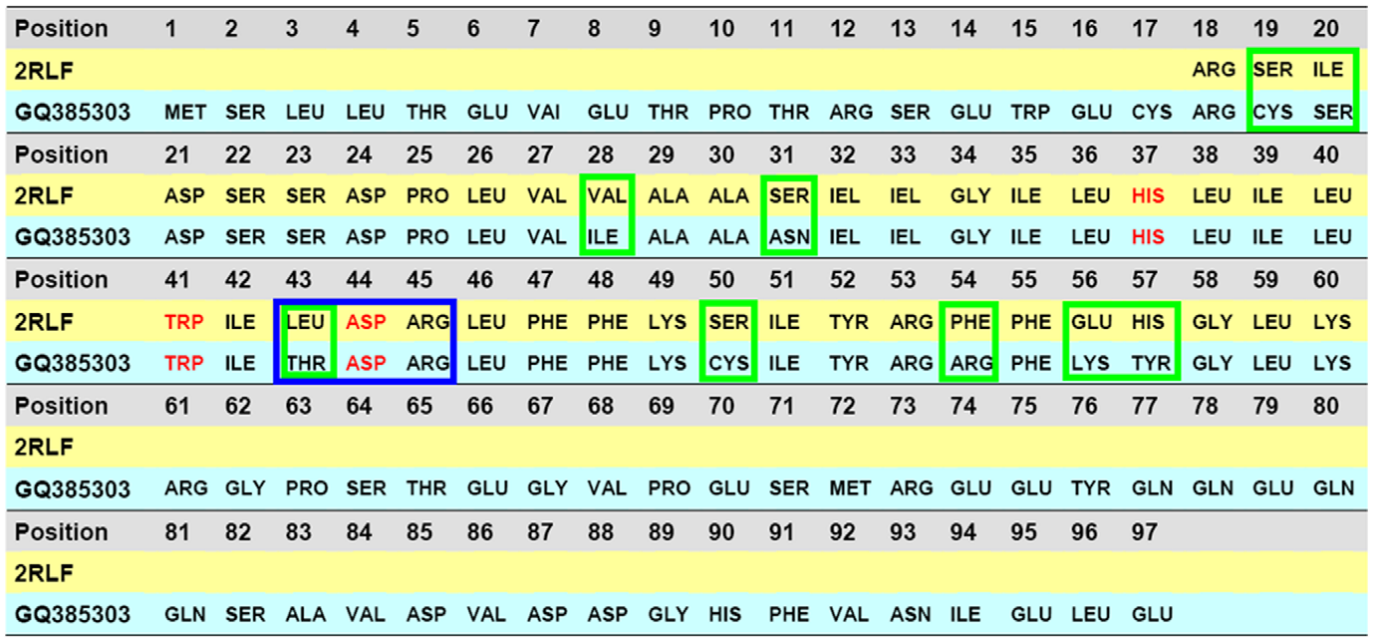
The sequence alignments between the M2 protein isolated from H1N1 influenza A virus (NCBI code: GQ385383) and the M2 channel isolated from the Udorn strain of human influenza virus. The latter 3D structure has been determined by NMR [16] with PDB code of 2RLF, and can be used to serve as a template to model the former. The GQ385383 is a complete M2 sequence consisting of 97 residues, while 2RLF only contains a segment of 43 structure-defined residues (18–60). The red codes highlight the functional residues: pH sensor His37, channel gate Trp41, and channel lock Asp44. Those residues, which are different between the two M2 sequences, are framed by a box drawn in green line, while those framed with a box drawn in light-blue line indicate the possible binding sites (Thr43, Asp44, and Arg45) of inhibitors. The functional residue Asp44 is the first binding site, and the Arg45 or Thr43 is the possible second binding site. Thr43 is a natural mutation Leu43Thr in the H1N1-M2 proton channel.

The sequence alignment is performed between the targeted protein (GQ385303) and the template (2RLF). The result of alignment is shown in **Fig. 1**, where the characters highlighted in red indicate the three functional residues (pH sensor His37, channel gate Trp41, and channel lock Asp44) [16]–[18], [21], [22] of the M2 channel, which are highly conserved in the M2 proteins. Those residues with the light-blue frame are the possible binding sites (Thr43, Asp44, and Arg45) of the inhibitors; while those with the green frame are different between the two sequences.

**Figure 2:**
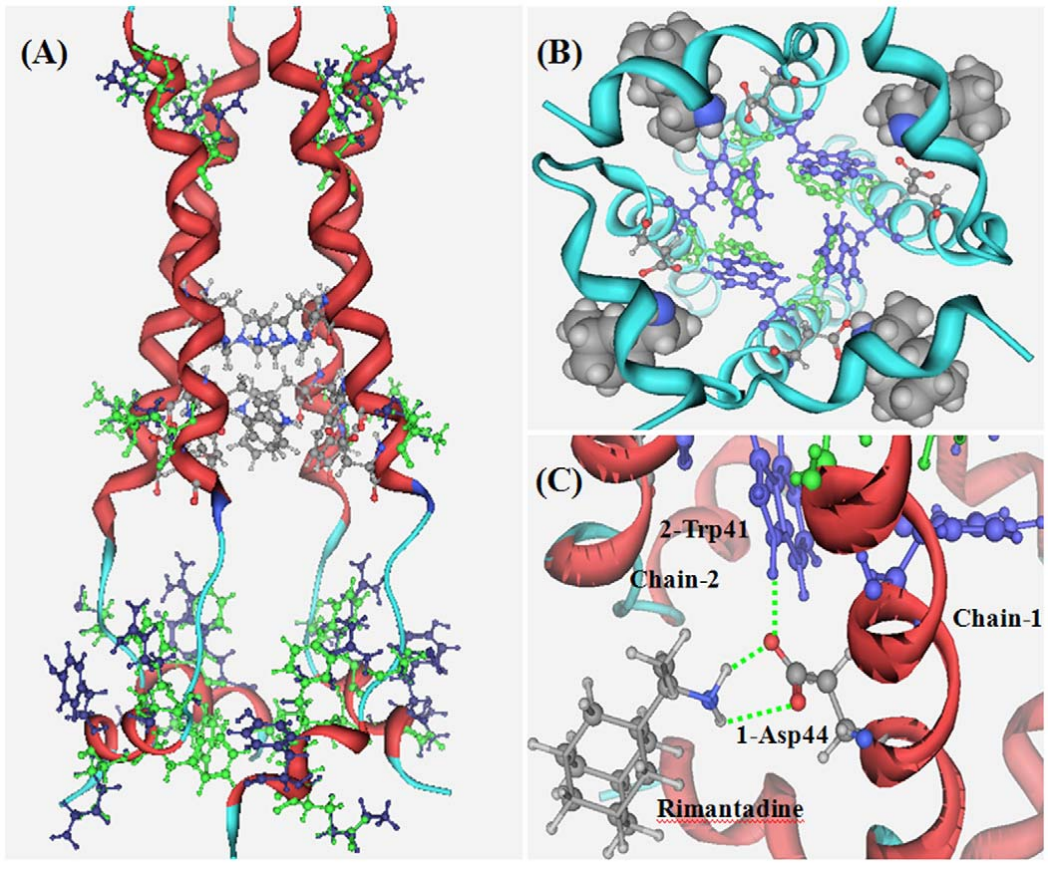
The computational three-dimensional structure of 2009-H1N1 M2 proton channel (NCBI code: GQ385303) and the template 2RLF with ligand rimantadine. (**A**) Superposition of the homology model of 2009-H1N1 M2 proton channel with its template, the NMR structure with the PDB code of 2RLF [16]. The two M2 proteins are highly homologous, and no differences are found on their backbones. The three functional residues of the M2 channel are rendered in ball-and-stick drawing with chemical element color; they are located at the middle of the channel. The different residues between two M2 proteins are show in green for 2RLF and blue for GQ385383. (**B**) A close-up view from the bottom of the M2 channel. The three functional residues are rendered in green for pH sensor His37, blue for channel gate Trp41, and chemical element color for channel lock Asp44. The ligand rimantadine binds at four equivalent sites near the channel lock Asp44 on the lipid-facing side of the channel and stabilizes the closed conformation of the pore. (**C**) The complex structure between the inhibitor rimantadine and the receptor M2 proton channel. The channel lock Asp44 holds the channel gate Trp41 through a hydrogen bond keeping it in the closed conformation. The ligand rimantadine forms two hydrogen bonds (green dotted lines) with the Asp44 of M2 proton channel.

Thus, using the structural bioinformatics tools [23], the 3D structure of the H1N1-M2 (GQ385383) channel was developed by following the same procedures as elaborated in references [24]–[26]. The computed 3D structure of H1N1-M2 channel (GQ385383) is shown in **Fig. 2A**. Subsequently, the Auto Dock program [27] with the MMFF94 force field and atomic partial charges [28] was utilized to dock the ligands to the H1N1-M2 channel. The starting point of the docking calculations was the position at which the drug rimantadine is located in the NMR structure of 2RLF [16]. A cubic box with side length of 30 Å surrounding the ligand was used for the docking calculations. A total of 25 docking conformations with the lowest binding energies were recorded.

## Results

Before the 3D NMR structure of M2 protein [16] was published, it was traditionally thought that the adamantane-based inhibitors were bound inside the channel and hence physically blocking the pore of channel [6]. With the novel allosteric inhibition mechanism revealed by the NMR structure [16], we can conduct the structure-based inhibitor design and find effective drugs against the H1N1-M2 channel in a completely different strategy [19], [29], as illustrated below.

### Computed Structure of H1N1 M2 Proton Channel

By following the procedures as described in the Materials and Methods section, the 3D structure of H1N1-M2 (GQ385383) was developed as shown in **Fig. 2A**, where for facilitating comparison the template structure is also given in a superposition manner with the targeted structure. The structural data can be provided by authors upon inquire. As we can see from the figure, the backbones of the two structures are almost the same as expected because the sequences of the two proteins are highly homologous. The three functional residues (His37, Trp41, and Asp44) of the M2 channel are rendered in ball-and-stick drawing with the chemical element color. They are located at the middle of the channel. The different residues between the two channel structures are shown in green for 2RLF and blue for GQ385383. Most mutated residues in H1N1-M2 protein are at the top and bottom of the channel, and they have little effects on the functional residues (His37, Trp41, and Asp44). However, one mutation, namely Leu43Thr, is found around the active region: the non polar residue Leu43 of 2RLF is replaced by the polar residue Thr43 in the H1N1-M2 protein. A close-up view from the bottom of the M2 channel is given in **Fig. 2B** to show the position and orientation of the three functional residues and the position of ligand rimantadine. The three functional residues are rendered in green for pH sensor His37, blue for channel gate Trp41, and chemical element color for channel lock Asp44, respectively. Ligand rimantadine binds at four equivalent sites near the channel lock Asp44 on the lipid-facing side of the channel. The Asp44 residue is not only the channel lock, but also the proton exit of the M2 channel. Therefore, drug binding at this position not only can hold the channel in the closed conformation but also can simply block the proton exits. [22].

Illustrated in **Fig. 2C** is the gating and inhibiting mechanism of the M2 proton channel [16]–[18], [22]. The channel lock Asp44 holds the channel gate Trp41 through a hydrogen bond, keeping it in the closed conformation in the middle or higher pH (∼7.5) condition. But in the lower pH environment, the pH sensor His37 and the indole amine of Trp41 are protonated, so as to weaken the hydrogen bond between Trp41 and Asp44, making it easily broken by the repulsive interaction between the positively charged His37 residues of two adjacent helix chains. After rimantadine binds at the Asp44 through two hydrogen bonds between amino group of rimantadine and carboxyl group of Asp44, the pK_a_ value of Asp44 is lowered by the two hydrogen bonds [30], [31]. Therefore, in the lower pH environment, it becomes difficult for Asp44 to be protonated. That is why in the acidic condition the ligand rimantadine can help Asp44 to keep the channel in the closed conformation.

### Inhibitors with Two Pharmacophore Groups

**Figure 3:**
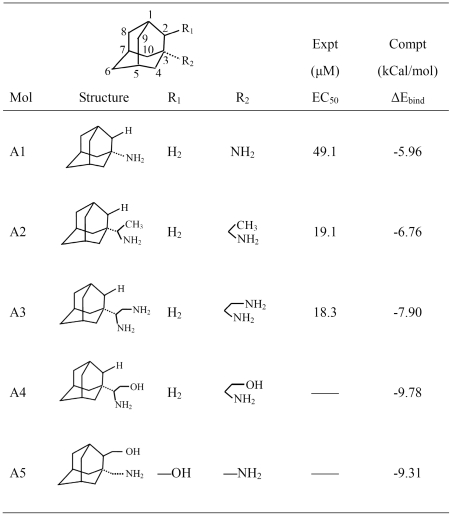
Summary of the existing adamantane-based drugs and newly designed inhibitors with two pharmacophore groups. A1 and A2 are the commercially available drugs amantadine and rimantadine. A3 was reported in ref [13]. A4 and A5 are inhibitors with two pharmacophore groups designed in this study.

Almost all existing M2 channel inhibitors have only one pharmacophore group, and in most cases it is an amino group [19]. The structures of several adamantane-based drugs are shown in **Fig. 3**, where the inhibitors A1 and A2 are commercially available drugs amantadine and rimantadine, respectively, in which the pharmacophore substitutes are at the position 3 of adamantine [13], [32], [33]. Actually, the pharmacophore substitutes also can be put on the position 2 of adamantine [11], [12], [19], and in many cases the substitutes on position 2 can give better results than on the position 3 [19].

**Figure 4:**
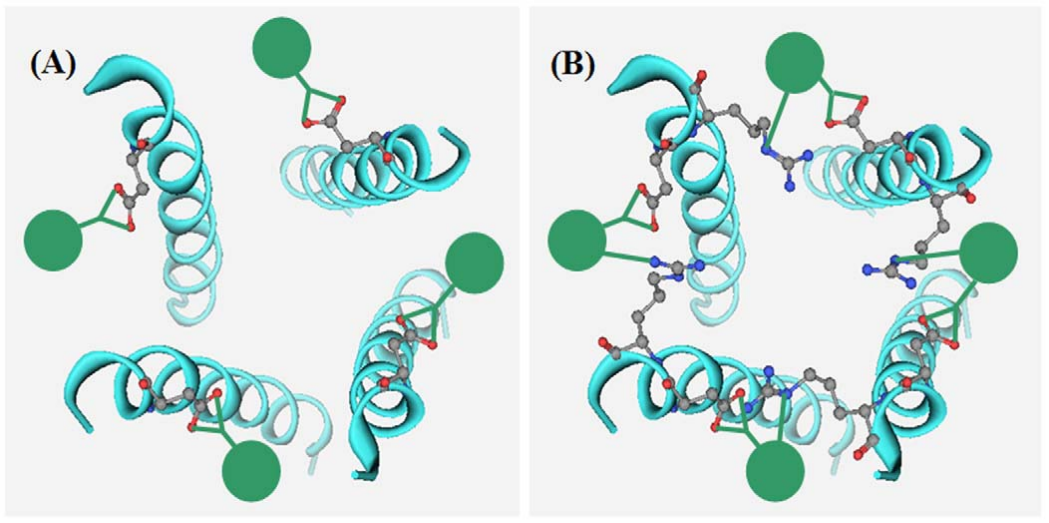
The binding models between M2 proton channel and adamantane-based inhibitors with one and two pharmacophore groups. (**A**) The adamantane-based inhibitors with one pharmacophore group can bind on only one helix of the tetrameric M2 channel. (**B**) With an additional pharmacophore group added into the adamantane-based inhibitors, the inhibitors can bind at the two sites of two neighboring helices of the M2 channel, and hence can hold the M2 channel in closed conformation more effectively, just like a barrel hoop. Actually only one such inhibitor is sufficient to block the M2 channel because it can block half of the channel and stop the conductance of proton (H_3_O^+^) flow effectively.

The M2 channel is a tetramer consisting of four identical helices. As shown in **Fig. 4A**, the adamantane-based inhibitor with one pharmacophore group can only bind on one helix of the tetrameric M2 channel. If an additional pharmacophore group is added into the adamantane-based inhibitor, it will be able to bind at two sites of two neighboring helices. Thus, the inhibitor will be able to hold the M2 channel in the closed conformation with a more effective way, just like a hoop in fastening a barrel, as shown by **Fig. 4B**. Actually only one such inhibitor molecule would be sufficient to block the M2 channel because the hoop-like inhibitor can block half of the channel and stop the conductance of proton (H_3_O^+^) flow.

### Structure-Based Inhibitor Design for M2 Protein

The discussions for the adamantane-based inhibitors with more than one pharmacophore groups can be found in references [12], [13]. However, before a reliable 3D structure of M2 channel is available, this kind of design was lack of a footing since we did not know the binding site and the interaction mechanism of the second pharmacophore. The inhibitor A3 in **Fig. 3** has two amino groups and possesses the bioactivity slightly higher than that of rimantadine [12], [13]. However, based on our docking studies, the A3 inhibitor could not effectively bind at the two neighboring helices. The inhibitor with two pharmacophore groups needs two binding sites on the M2 channel. The first binding site is unchangeably the carboxyl group of the Asp44, while the second binding site could be either the amino group of Arg45 or the hydroxyl group of Thr43 of the neighboring helix. These two residues are the closest residues to the first binding site Asp44.

**Figure 5:**
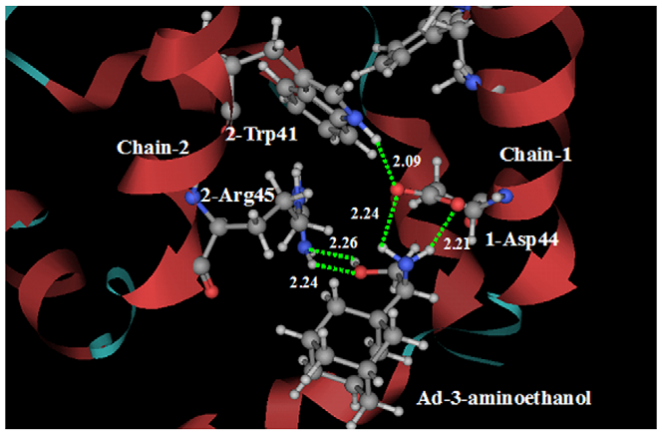
Illustration to show the interactions between the designed inhibitor A4 (Ad―3―aminoethanol) and the M2 proton channel. The designed adamantane-based inhibitor A4 has two pharmacophore groups. The amino group binds at the 1-Asp44 of Chain-1 through two hydrogen bonds (green dotted lines), while the second pharmacophore hydroxyl group forms two hydrogen bonds with the 2-Arg45 of Chain-2. It is through the two binding sites that the inhibitor A4 holds the Chain 1 tightly with its adjacent Chain 2 of the tetrameric M2 proton channel.

With two pharmacophore groups, the inhibitors A4 and A5 in **Fig. 3** were designed based on the structure of H1N1-M2 proton channel. On the subsite of inhibitor A4 corresponding to the position 3 of adamantine, there is an amino group and a hydroxyl group. Docking calculation gives an illustration for the interactions between the designed inhibitor A4 and the M2 proton channel. The amino group of inhibitor A4 binds at the 1-Asp44 of Chain-1 through two hydrogen bonds, while the second pharmacophore hydroxyl group forms two hydrogen bonds with the amino group of 2-Arg45 of Chain-2. The detailed interactions between M2 channel and the ligand A4 are shown in **Fig. 5**. It is through the two binding sites that the inhibitor A4 holds the Chain-1 tightly with its adjacent Chain-2 of the tetrameric M2 proton channel. Actually, the inhibitor A4 was firstly synthesized by Clariana et al. in 2000 [34] not as an anti flu drug, but as a reagent for studying peptide–receptor interactions. As for inhibitor A4, no bioactivity data to M2 proton channel was reported.

**Figure 6:**
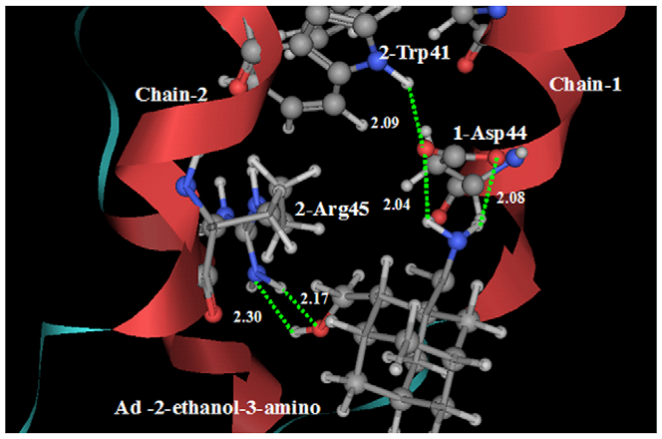
Illustration to show the interactions between the designed inhibitor A5 (Ad―(2―ethanol)―(3―amino)) and the M2 proton channel. The designed adamantane-based inhibitor A5 has two pharmacophore groups. The amino group of inhibitor A5 binds at the 1-Asp44 of Chain-1 through two hydrogen bonds (green dotted lines), while the second pharmacophore hydroxyl group forms two hydrogen bonds with the 2-Arg45 of Chain-2. It is through the two binding sites that the inhibitor A5 holds the Chain-1 tightly with its adjacent Chain-2 of the tetrameric M2 proton channel.

The designed inhibitor A5 has two pharmacophore groups: a hydroxyl group on position 2 and an amino group on position 3 of adamantane. Illustrated in **Fig. 6** is a close view of the interactions between the designed inhibitor A5 and the M2 proton channel. The amino group of inhibitor A5 binds at the carboxyl group of 1-Asp44 of Chain-1 through two hydrogen bonds, while the second pharmacophore hydroxyl group forms two hydrogen bonds with the amino group of 2-Arg45 of Chain-2. Therefore, it is in the same way as inhibitor A4 that the inhibitor A5 holds the Chain 1 tightly with its adjacent Chain 2 of the tetrameric M2 proton channel.

### Energetic Analysis for M2 Channel-Inhibitor Interactions

During the molecular docking of the inhibitors to the tetrameric M2 proton channel, a total of 25 conformations with the strongest binding energies were recorded for each of the five inhibitors in **Fig. 3**, where the most favorable binding energy for each of the five inhibitors are also given. It can be seen from the **Fig. 3** that ΔE_A1_<ΔE_A2_ <ΔE_A3_<<ΔE_A5_<ΔE_A4_, indicating that the binding free energies of A4 and A5 are much stronger than those of A1 and A2 due to the contributions of the additional pharmacophore, namely the hydroxyl group, which forms two additional hydrogen bonds with the receptor. Although A3 inhibitor also possesses two amino groups and the second amino group can form hydrogen bonds with the amino group of Arg45, the hydrogen bonds between the two amino groups are easily broken in acidic condition.

## Discussion

The NMR structure of M2 proton channel [16] provides a reliable structural basis for rational drug design against influenza virus. The channel gating mechanism and the inhibiting mechanism of M2 proton channel, revealed by the NMR structure of M2 proton channel, stimulate the new idea and strategy for channel inhibitor design. The two inhibitors (A4 and A5) of M2 proton channel, designed in this study, are hopefully the potential drugs for the 2009-H1N1 swine flu.

The adamantane-based drugs (amantadine and rimantadine) are not like the ordinary drug molecules from the viewpoint of Lipinski's “the rule of five” [35], a rule of thumb to evaluate druglikeness, or to determine if a chemical compound with a certain pharmacological or biological activity has properties that would make it a likely orally active drug in humans. This is because amantadine and rimantadine possesses very few pharmacophore groups and very few hydrogen-bond-forming elements. Rimantadine can hold one of the four helices in the tetrameric M2 proton channel by one, and only one, pharmacophore amino group. An additional pharmacophore group is needed to hold the adjacent helix of the tetrameric channel so as to strengthen its closed conformation for blocking the proton conductance. The high-resolution NMR structure of the M2 proton channel [16] and the gating and inhibiting mechanism revealed therefrom has made it possible to rationally design new and more powerful drugs against influenza viruses. It is the second pharmacophore group in the inhibitors A4 and A5 that might significantly enhance their ability in inhibiting the M2 channel in comparison with amantadine and rimantadine.

The M2 proton channel is a membrane protein; while the adamantane-based inhibitors are detergent-like compounds with a hydrophilic head and a hydrophobic body, possessing the ability to penetrate the bilayer lipid membrane. Although the second additional hydrophilic pharmacophore group of A4 and A5 inhibitors can enhance their inhibition ability to the M2 proton channel, it might lower their ability in penetrating membrane. Therefore, a series of follow-up experiments are needed along this direction to find an optimal inhibitor by taking into account these two aspects.
